# Associations between depressive symptoms and geriatric syndromes in community-dwelling older adults in Japan: A cross-sectional study

**DOI:** 10.1016/j.pmedr.2021.101353

**Published:** 2021-03-10

**Authors:** Masakazu Imaoka, Hidetoshi Nakao, Misa Nakamura, Fumie Tazaki, Mitsumasa Hida, Tomoko Omizu, Ryota Imai, Masatoshi Takeda

**Affiliations:** a Department of Rehabilitation, Osaka Kawasaki Rehabilitation University, 158 Mizuma, Kaizuka, Osaka 597-0104, Japan; b Cognitive Reserve Research Center, 158 Mizuma, Kaizuka, Osaka 597-0104, Japan; c Department of Comprehensive Rehabilitation, Osaka Prefecture University, 3-7-30, Habikino, Habikino, Osaka 583-8555, Japan; d Department of Preventive Gerontology, National Center for Geriatrics and Gerontology, 7-430 Morioka, Obu, Aichi 474-8511, Japan; e Department of Rehabilitation, Kansai University of Welfare Sciences. 3-11-1 Asahigaoka, Kasihara, Osaka 582-0026, Japan

**Keywords:** AWGS, Asian Working Group for Sarcopenia, MMSE, Mini-Mental State Examination, OR, Odds ratio, Depressive symptoms, Frailty, Sarcopenia, Locomotive syndrome, Older adults

## Abstract

It is estimated that 7.2% of community-dwelling older adults worldwide have major depression. Therefore, this study aimed to investigate the relationship between geriatric syndromes and depressive symptoms. Data were obtained from the Kaizuka Dementia Prevention Study 2018 and 2019, which was a community-based health check conducted in collaboration with the Osaka Kawasaki Rehabilitation University (Kaizuka City Office) and Cognitive Reserve Research Center in Osaka, Japan. The participants comprised 363 older adults (mean age 73.6 ± 6.6 years; women = 75.8%) who participated in a community-based health check. Depressive symptoms were assessed using the 15-item Geriatric Depression Scale (GDS-15). Depressive symptoms were defined as a GDS-15 score of ≥ 5. Furthermore, geriatric syndromes in participants—such as frailty, sarcopenia, and locomotive syndrome—were assessed. There was a 28.1% prevalence of depressive symptoms. In a logistic regression analysis with depressive symptoms as the dependent variable, both pre-frailty (odds ratio [OR] 1.85, 95% confidence interval [CI] 1.09–3.01) and frailty (OR 5.45, 95% CI 2.23–13.31) were found to be significantly higher in the depressive group. There were no significant differences in sarcopenia and locomotive syndrome between the depressive groups. Our findings suggest that depressive symptoms are associated with frailty and pre-frailty in community-dwelling older adults in Japan. Physical frailty should be evaluated in depressed individuals and may contribute to the prioritization of clinical evaluation of geriatric syndromes.

## Highlights

• Depressive symptoms are associated with frailty in community-dwelling older adults.
• AWGS’s new sarcopenia definition is not associated with depressive symptoms.
• Depressive symptoms may be associated with J-CHS-defined frailty.
• Among geriatric syndromes, only frailty may be associated with depressive symptoms.

## 1 Introduction

Depression is a common psychiatric disorder that affects approximately 322 million people worldwide (World Health Organization, 2017). It is estimated that 7.2% of community-dwelling older adults worldwide have major depression, and 17.1% have had depression throughout their lives (Luppa et al., 2012). Factors associated with depression in old age include deterioration of life functions (Iwasa et al., 2009) and decline in physical (Hu et al., 2019) and cognitive functions (Potvin et al., 2011). Other factors related to living conditions, such as living alone and being divorced, have also been reported (Weyerer et al., 2008). Depressive symptoms are associated with adverse health outcomes, including risk of stroke and all-cause mortality (Eurelings et al., 2018).

Previous studies with community-dwelling older adults have reported associations between depressive symptoms and physical (Yoshida et al., 2015) and cognitive functions (Livingston et al., 2017). In a three-year prospective cohort study with 680 community-dwelling older adults by Yoshida et al. (2015), continued physical activity in old age was the only factor associated with a reduction in the incidence of depressive symptoms. Another study identified depression as a risk factor for dementia (Livingston et al., 2017). To date, depressive symptoms have been evaluated from the perspective of exercise or cognitive function alone, but not both.

Recent geriatric syndrome studies addressed several complex conditions associated with aging, such as depression, frailty (Fried et al., 2001), sarcopenia (Chen et al., 2014), and locomotive syndrome (Muranaga and Hirano, 2003). One study reported that community-dwelling frail older adults are at high risk of depression (Makizako et al., 2015). A systematic review reported an independent association between depression and sarcopenia in community-dwelling older adults (Chang et al., 2017), and a cross-sectional study reported an association between depression and locomotive syndrome (Muranaga and Hirano, 2003).

Although the association between these geriatric syndromes and depression is clear, the strength of the association is not evident. In addition, these definitions of geriatric syndrome are operational, and it is necessary to carefully examine their relevance to the latest definitions. Therefore, we decided to use the latest definition of geriatric syndrome to determine its association with depressive tendencies.

Our findings may contribute to the prioritization of the clinical evaluation of geriatric syndromes and highlight the importance of focusing on the items included in geriatric syndromes, such as muscle strength, muscle mass, mobility, fatigue, and weight loss.

## 2 Materials and methods

### 2.1 Aim, design, and setting

Data from the Kaizuka Dementia Prevention Study 2018 and 2019 were used in this cross-sectional study. The study was a community-based health check conducted in collaboration with the Osaka Kawasaki Rehabilitation University (Kaizuka City Office) and Cognitive Reserve Research Center in Osaka, Japan.

### 2.2 Study population

**Fig. 1:**
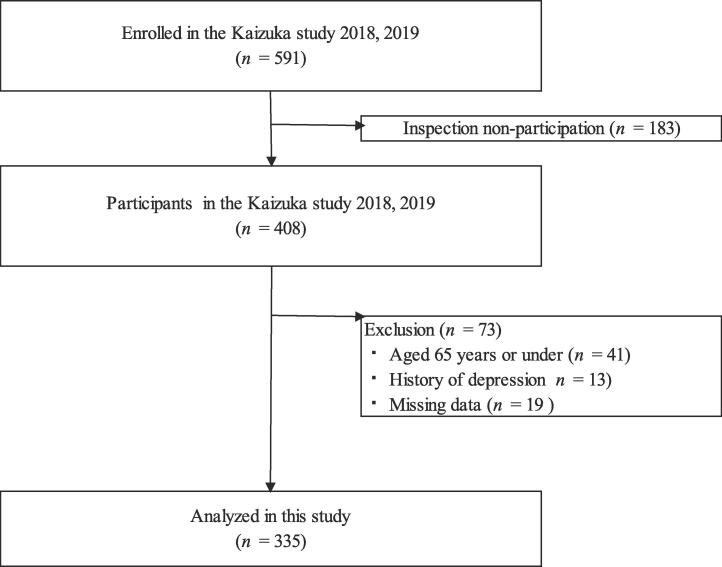
Flow chart of the study.

Participants were residents of Kaizuka City and aged ≥ 65 years at the time of the study. To recruit participants, we inserted leaflets in newspapers, posted notices at the city hall, and accepted applications over the phone. Exclusion criteria were a history of depression (*n* = 13), aged 65 years or under (*n* = 41), and incomplete data on the survey (*n* = 19). Data from 335 participants (mean age 74.8 [SD ± 5.7] years; women *n* = 252 [75.2%]) were analyzed (Fig. 1).

### 2.3 Ethical considerations

This study was approved by the Ethics Committee of the Osaka Kawasaki Rehabilitation University (Reference No. OKRU29-A016). Written informed consent was obtained from all participants in accordance with the tenets of the Declaration of Helsinki. No compensation was provided for participation.

### 2.4 Measures

#### 2.4.1 Depressive symptoms

We used the Japanese version of the Geriatric Depression Scale 15 (GDS-15) to determine the existence of depressive symptoms among the participants. The GDS-15 is a self-report instrument comprising 15 items that assess the individual’s mood (Sugishita et al., 2017). A cut-off point of 4/5 was used to define depressive symptoms in this study.

#### 2.4.2 Body muscle mass

We collected data on physiological parameters obtained using bioelectrical impedance analysis (InBody270; InBody, Tokyo, Japan) at 20 and 1000 kHz frequencies from participants’ electronic medical records (Nakamura et al., 2017). Measurements were recorded within a 30-second period by staff. Body mass index was calculated by dividing body weight (kg) by squared height (m^2^). The appendicular skeletal muscle index was derived from the appendicular muscle mass (kg) divided by squared height (m^2^). Low muscle mass was defined as a height-adjusted skeletal muscle mass <7.0 kg/m^2^ for men and <5.7 kg/m^2^ for women.

#### 2.4.3 Muscle strength

Muscle strength was measured by handgrip strength, which has been reported to be significantly associated with whole-body muscle strength (Makizako et al., 2017). The maximum voluntary isometric strength of the handgrip was measured using a Grip-D hand dynamometer (Takei; Niigata, Japan) for the dominant hand while in a standing position. Low muscle strength was defined as a handgrip strength <28 kg in men and <18 kg in women.

#### 2.4.4 Gait speed

We instructed participants to walk 6.4 m (divided into two 2-m zones at each end and a 2.4-m zone in the middle) at a speed they found comfortable. The time needed (s) to pass the 2.4-m middle zone was measured to calculate the gait speed (m/s) (Shimada et al., 2013). Participants could use a cane or walker if they were unable to walk without help. We used the average of five gait trials.

#### 2.4.5 Cognitive status

We used the Japanese version of the Mini-Mental State Examination (MMSE), which is widely used to assess cognitive function and screen for cognitive impairment (Folstein et al., 1975). MMSE scores range from 0 to 30, with lower scores representing weaker cognitive function.

#### 2.4.6 Locomotive syndrome

We measured locomotive syndrome using the two-step test. Participants were asked to take two steps forward as far as possible and stop after the second step (Muranaga and Hirano, 2003). Participants were retested if they lost balance during the test or if they did not stop after the second step. They were not allowed to jump. The test was carried out until two results were obtained. The maximum length (m) covered in the two tests was selected and divided by the participant’s height (m). Results were categorized as robust (≥1.3 m/m), Locomo1 (<1.3 m/m), and Locomo2 (<1.1 m/m), based on the Japanese Orthopedic Association criteria.

#### 2.4.7 Sarcopenia

We determined participants’ sarcopenia status using the Asian Working Group for Sarcopenia (AWGS) consensus recommendations (Chen et al., 2019) in terms of the results of the gait test, muscle strength, and muscle mass. Participants with skeletal muscle mass loss (<7.0 kg/m^2^ for men and <5.7 kg/m^2^ for women), low physical function (low grip strength [<28 kg for men and <18 kg for women] and slowness, indicated by normal walking speed [<1.0 m/sec]), and skeletal muscle mass loss without low physical function were determined as having pre-sarcopenia.

#### 2.4.8 Frailty

The characteristics of frailty used in this study (Fried et al., 2001) have been described in detail elsewhere. The components of frailty as defined by Fried et al. (2001) are as follows: Weight loss, weakness, exhaustion, low activity level, and slowness. Weight loss was determined using the question “Have you lost 2 kg or more in the past 6 months?” taken from the Kihon Checklist, a self-report, comprehensive health checklist. Exhaustion was determined based on a response of “yes” to another question from the Kihon Checklist, “In the last 2 weeks, have you felt tired for no reason?” (Fukutomi et al., 2015). Weakness was defined as low muscle strength based on the grip strength assessment described earlier. Low activity level was defined as a negative response to the following questions: “Do you engage in moderate levels of physical exercise or sports aimed at health?” and “Do you engage in low levels of physical exercise aimed at health?” Slowness was defined as a walking speed of <1.0 m/s. Participants were classified as “robust” if they had none of these symptoms, “pre-frail” if they had one or two symptoms, and “frail” in the case of three or more symptoms (Shimada et al., 2016).

#### 2.4.9 Covariates

We collected data on sociodemographic variables, including age and gender. The covariates in this study comprised these sociodemographic variables, as well as cognitive function and physical function.

### 2.5 Statistical analysis

All analyses were performed using IBM SPSS Statistics version 24.0 (IBM Corp., Armonk, NY, USA). *P*-values < 0.05 were statistically significant. To compare the depressive and non-depressive groups, we used Pearson’s χ^2^ tests for categorical variables, Student’s *t*-test for continuous variables, and the Mann–Whitney *U* test for ordinal variables. Next, a multivariate analysis was performed on the items of geriatric syndrome that showed significant differences in the univariate analysis. Non-linear logistic regression analyses were performed to examine the association between geriatric syndrome and depressive symptoms. There were two regression models: one used crude odds ratios (ORs), while the other used adjusted age and gender ORs. Presence of depressive symptoms was the dependent variable in both models.

## 3 Results

The participant characteristics are shown in Table 1. Of the 363 participants, 102 (28.1%) had some depressive symptoms, as determined by their GDS-15 scores. Participants in the depressive group were more likely to be frail or pre-frail and had lower MMSE scores and a higher frailty rate than those in the non-depressive group.

**Table 1:** Characteristics of the participants.

Variable	All participants (*n* = 335) mean ± SD			Non-depressive group (*n* = 239) mean ± SD			Depressive group (*n* = 96) mean ± SD			*p-*value
Age, years	74.8	±	5.7	74.7	±	5.6	75.1	±	6.3	0.59
Female sex, *n* (%)	252	(75.2)		179	(74.9)		73	(76.0)		0.89
Grip strength, kg	23.5	±	6.9	23.8	±	7.0	23.0	±	6.6	0.30
Gait speed, m/s	1.3	±	0.2	1.3	±	0.2	1.3	±	0.2	0.60
SMI, kg/m^2^	6.0	±	0.9	6.1	±	0.9	6.0	±	0.9	0.40
MMSE score	28.3	±	2.4	28.5	±	2.2	27.9	±	2.7	0.04
GDS-15 score	3.3	±	2.4	2.1	±	1.3	6.3	±	1.4	0.00
Frailty, *n* (%)										
Robust	139	(41.5)		110	(46.0)		29	(30.2)		0.00
Pre-frail	169	(50.4)		118	(49.4)		51	(53.1)		
Frail	27	(8.1)		11	(4.6)		16	(15.2)		
Locomotive syndrome, *n* (%)										
Robust	78	(23.3)		55	(23.0)		23	(24.0)		0.43
Locomo1	150	(44.8)		112	(46.9)		38	(39.6)		
Locomo2	107	(31.9)		72	(30.1)		35	(36.5)		
Sarcopenia, *n* (%)										
Robust	275	(82.1)		198	(82.8)		77	(80.2)		0.85
Sarcopenia	44	(13.1)		30	(12.6)		14	(31.8)		
Severe sarcopenia	16	(4.8)		11	(4.6)		5	(5.2)		

SD: standard deviation, SMI: Skeletal Muscle Mass Index,

MMSE: Mini-Mental State Examination, GDS-15: Geriatric Depression Scale

Table 2 shows the results of the univariate and multivariate logistic regression analyses. The univariate logistic regression analysis showed that pre-frailty (OR 1.64, 95% confidence interval [CI] 0.97–2.77) was not related to depressive symptoms, whereas frailty (OR 5.52, 95% CI 2.31–13.17) was related to depressive symptoms (Crude Model). A multivariate adjusted for potential covariates also showed that pre-frailty (OR 1.64, 95% CI 0.97–2.78) was related to depressive symptoms, whereas frailty (OR 5.71, 95% CI 2.35–13.89) was significantly related to depressive symptoms (Adjusted Model 1).

**Table 2:** Association between depressive symptoms and geriatric syndrome in study participants*.

Frailty	Crude OR*	95%CI	*p*-value	aOR†	95%CI	*p*-value
Robust	1.00			1.00		
Pre-frail	1.64	0.97–2.77	0.07	1.64	0.97–2.78	0.06
Frail	5.52	2.31–13.17	0.00	5.71	2.35–13.89	0.00

* Presence of depressive symptoms was the dependent variable, and frailty was an independent variable;

† Adjusted for age and gender.

## 4 Discussion

This cross-sectional study showed that frailty was significantly associated with depressive symptoms in older adults after adjusting for potential covariates. Thus, our results suggest that depressive symptoms are associated with frailty as defined by the Japan-Cardiovascular Health Study. However, other geriatric syndromes, specifically based on a new definition of sarcopenia and locomotive syndrome, were not associated with depressive symptoms.

A previous study reported a prevalence of depression among older adults at 23.6% (Pegorari and Tavares, 2014). In this study, the prevalence of depressive symptoms was 28.7%, which is significantly higher. It is possible that, because the participants in this study were recruited via city hall notices and newspaper leaflets, the participation rate of older adults with health anxiety was higher.

A study with 958 adults older than 60 years found that depressive symptoms had a significant association with frailty (OR 1.8), which supports our results (Feng et al., 2014).

Previous studies on frailty and depression as overlapping geriatric syndromes reported that the latent factors of frailty that were significantly correlated with depression were biological syndrome (ρ = 0.68, *p* < 0.01), functional domains (ρ = 0.70, *p* < 0.01), and frailty index (ρ = 0.61, *p* < 0.01) (Lohman et al., 2016). The biological syndrome model of frailty was operationalized in terms of five criteria proposed by Fried et al. (2001), who analyzed data from the Cardiovascular Health Study. The frailty index is a count of 70 clinical deficits, including presence of diseases, difficulty in performing daily activities, and other physical and neurological signs and symptoms. In terms of functional domains, frailty was defined as functional impairment in at least two of four domains: physical, nutritive, cognitive, and sensory. For example, a previous study (Mezuk et al., 2013) reported that models of frailty and depression with differing numbers of classes representing the severity of the syndromes (two in frailty; three in depression) provided a better fit to their data, although the constructs shared a substantial amount of variance (latent kappa coefficient = 0.66); moreover, these also helped identify overlapping subgroups of participants. Using confirmatory factor analysis in the Health and Retirement Study among 3453 participants, a moderate correlation (0.68) was found between latent categorical models of depression and the “biological definition” of frailty (Lohman et al., 2014). These findings suggest that pre-frailty and frailty are associated with depressive symptoms. Frailty comprises reduced motor functions such as walking speed and grip strength, and these can be improved by exercise therapy, even in older adults (Chan et al., 2012). Therefore, it may be possible to use non-pharmacological interventions to reduce depression in older adults.

Contrary to expectations, no geriatric syndromes other than frailty were associated with depressive symptoms. Although locomotive syndrome is an independent risk factor for depression (Muranaga and Hirano, 2003), in this study we found no significant difference between the non-depressive and depressive groups. The reason for this discrepancy may be that the GLFS-25 questionnaire includes items on psychological characteristics, such as pain and anxiety, which may be better measures of depressive symptoms.

Previous studies of sarcopenia (Chang et al., 2017) found a correlation between sarcopenia and depression; however, the present study did not find a significant correlation between the AWGS new sarcopenia definition and depressive symptoms. In a six-year prospective study with 1731 community-dwelling older adults, sarcopenia alone was not associated with depression but was associated with sarcopenia obesity (Ishii et al., 2016). Therefore, sarcopenia, which indicates muscle wastage, is not associated with depression alone, but it may be associated with depression regarding body mass index. Speed et al. (2019) investigated the relationship between fat weight, non-fat weight, and depression by analyzing the body composition data of 800,000 people and reported that fat weight was a risk factor for depression. The findings suggest that it is important to consider sarcopenia obesity and obesity, rather than sarcopenia alone, when assessing the risk of depression.

Our study had some limitations. First, we used screening tools rather than full diagnostic procedures to assess depressive symptoms. Second, the cross-sectional design of the study precluded the assumption of a causal relationship between depressive symptoms and frailty. Third, study data were obtained from a single city, which limits the generalizability of the results, as meaningful activities in daily life are also affected by geographic characteristics. Finally, the 335 older adults enrolled represented approximately 3% of older adults living in urban areas and were not randomly selected. Therefore, sampling bias cannot be ruled out.

## 5 Conclusions

This study found a significant association of frailty with depressive symptoms among community-dwelling older adults. Frailty is strongly associated with depressive symptoms, suggesting that improvement in frailty may alter depressive symptoms. We believe that this study may be useful in developing measures to reduce the risk of depressive symptoms among older adults.

## Availability of data and materials

The data that support the findings of this study are available from the Kaizuka Dementia Prevention Study Project Team; however, restrictions apply to the availability of these data, which were used under license for the current study, and are therefore not publicly available. Data are, however, available from the authors upon reasonable request and with permission from the Kaizuka Dementia Prevention Study Project Team.

## Funding

This research did not receive any specific grant from funding agencies in the public, commercial, or not-for-profit sectors.

## Authors’ contributions

HN, FT, MH, TO, and RI collected data, organized the dataset, and provided advice for consideration. MN contributed to the planning of research and survey items. TM organized the entire study. All authors read and approved the final manuscript.

## CRediT authorship contribution statement

**Masakazu Imaoka:** Conceptualization, Data curation, Formal analysis, Investigation, Project administration, Writing - review & editing. **Hidetoshi Nakao:** Data curation, Investigation. **Misa Nakamura:** Data curation, Formal analysis, Investigation, Supervision. **Fumie Tazaki:** Data curation, Investigation. **Mitsumasa Hida:** Data curation, Investigation. **Tomoko Omizu:** Data curation, Investigation. **Ryota Imai:** Data curation, Investigation. **Masatoshi Takeda:** Supervision.

## Declaration of Competing Interest

The authors declare that they have no known competing financial interests or personal relationships that could have appeared to influence the work reported in this paper.
